# Index-Catalogue Surgeon-General’s Office

**Published:** 1891-01

**Authors:** 


Index-Catalogue of the Library of the Surgeon-General's Office, United States Army. Compiled by John S. Billings, M. D., Surgeon U. S. Army. Authors and Subjects. Volume XI. Phædronus-Régent. Washington: Government Printing Office. 1890.

The present, or eleventh volume, of this remarkable work contains: Author-titles, 9,539, representing 4,535 volumes, and 8,908 pamphlets. It also contains 14,262 subject-titles of separate books and pamphlets, and 38,080 titles of articles in periodicals.

Year by year this stupendous labor goes on, and when completed will form the most comprehensive and useful reference catalogue the world has produced. Too much credit cannot be accorded to the compiler.

